# [Corrigendum] Associations between Huwe1 and autophagy in rat cerebral neuron oxygen-glucose deprivation and reperfusion injury 

**DOI:** 10.3892/mmr.2026.13833

**Published:** 2026-02-26

**Authors:** Guo-Qian He, Yan Chen, Hui-Juan Liao, Wen-Ming Xu, Wei Zhang, Guo-Lin He

Mol Med Rep 22: 5083–5094, 2020; DOI: 10.3892/mmr.2020.11611

Following the publication of the above article, the authors have contacted the Editor to explain that, regarding the immunofluorescence experiments shown in Fig. 3 on p. 5089, the data correctly shown in Fig. 3I (for the ‘V-ctrl+DMSO+R 24 h’ experiment) had inadvertently also been included for Fig. 3E (showing the result of the ‘DMSO+R 4 h’ experiment).

However, the authors had retained their original data, and were able to determine how this error had occurred. A revised version of Fig. 3, now showing the correct data for Fig. 3E, is shown on the next page. Note that the error made in assembling the data in Fig. 3 did not significantly affect either the results or the conclusions reported in this paper. All the authors agree with the publication of this Corrigendum, and are grateful to the Editor of *Molecular Medicine Reports* for allowing them the opportunity to publish this; moreover, they apologize to the readership for any inconvenience caused.

## Figures and Tables

**Figure 3.**
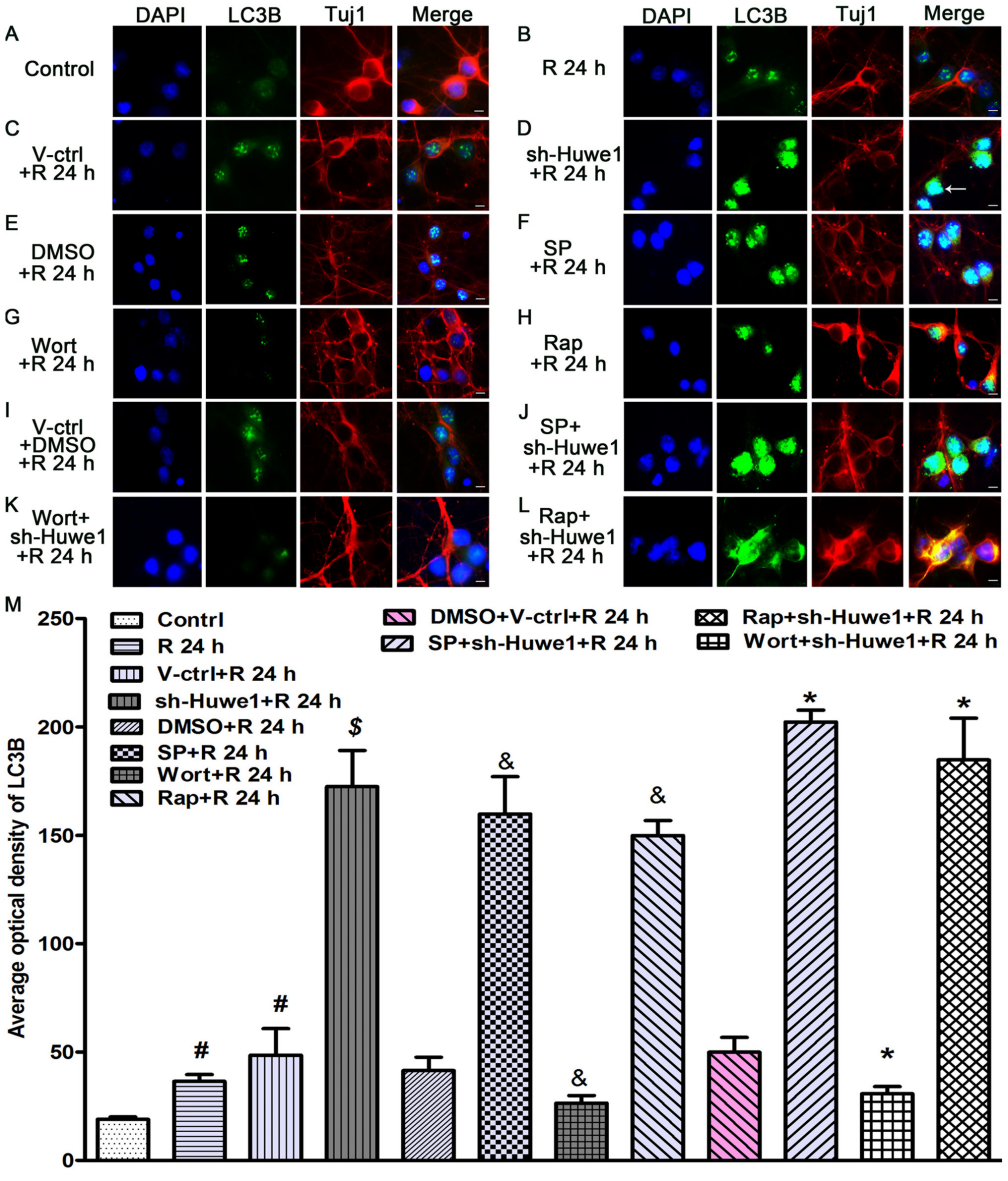
Immunofluorescence staining results for Tuj1 and LC3B. Cultured cortical neurons were infected with Huwe1 shRNA with or without an autophagy inhibitor, an autophagy inducer or a JNK pathway inhibitor, and then subjected to OGD/R for 24 h. Neuronal cells were stained with LC3B (green) and Tuj1 (red) for a double label immunofluorescence assay. DAPI (blue) was used to stain the cell nuclei. (A) Healthy, control neurons had normal polarity, with most of the cells having a single axon and multiple dendrites. LC3B was mainly localized to the cell nucleus in (B) cortical neuronal cells under OGD/R conditions compared with healthy control neurons. The cell bodies and proximal end of neurites became shortened and degraded at 24 h after OGD. (C) Compared with the control lentivirus, (D) treatment with Huwe1 shRNA increased the expression of LC3B. (E) Compared with DMSO, (F) the JNK inhibitor or (H) an autophagy inducer increased the expression of LC3B, as well as shortened neuronal axons at 24 h after OGD. (I) Compared with cotreatment with the control lentivirus and DMSO, (J) cotreatment with Huwe1 shRNA and a JNK inhibitor also increased the level of LC3B at 24 h. Compared with (E) DMSO, (G) Wort decreased the expression of LC3B and slightly reduced neurite degradation at 24 h after OGD. Relatively intact structure and polarity of some neurons was observed at 24 h upon (K) cotreatment with Huwe1 shRNA and Wort. (L) Cotreatment with Huwe1 shRNA and autophagy inducer also increased the level of LC3B at 24 h. Scale bar, 10 µm (magnification, ×60). (M) Quantitation of the staining results. ^#^P<0.05 vs. control group; ^$^P<0.05 vs. V-ctrl group; ^&^P<0.05 vs. DMSO group; *P<0.05 vs. the DMS + V-ctrl group. Wort, wortmannin; V-ctrl, green fluorescent protein-scramble control lentivirus-treated group; R, reperfusion; OGD, Oxygen-glucose deprivation; sh, short hairpin RNA; Huwe1, HECT, UBA and WWE domain containing E3 ubiquitin protein ligase 1; LC3, microtubule associated protein 1 light chain 3 α; Tuj1, neuron-specific class III β-tubulin; SP, SP600125; Rap, Rapamycin.

